# Synthesis of Hydroxyapatite/Iron Oxide Composite and Comparison of Selected Structural, Surface, and Electrochemical Properties

**DOI:** 10.3390/ma15031139

**Published:** 2022-02-01

**Authors:** Adrianna Biedrzycka, Ewa Skwarek, Dariusz Osypiuk, Beata Cristóvao

**Affiliations:** Faculty of Chemistry, Maria Curie-Sklodowska University in Lublin, Sq. Maria Curie-Skłodowska 2, PL-20031 Lublin, Poland; a.biedrzycka@poczta.umcs.lublin.pl (A.B.); dariusz.osypiuk@mail.umcs.pl (D.O.); beata.cristovao@mail.umcs.pl (B.C.)

**Keywords:** hydroxyapatite, iron oxide, composite, co-precipitation method

## Abstract

The paper presents the synthesis of a hydroxyapatite/iron oxide composite utilizing the wet chemical method, as well as the comparison of several selected material characteristics. As follows from the literature reports, hydroxyapatite is a common mineral possessing numerous significant properties. Nowadays, there is an increase in the amount of research on possible modifications of this compound. The promising way to improve hydroxyapatite features is its combination with iron oxide. Particularly, there can be two forms that are distinguished, namely Fe_3_O_4_ and γ-Fe_2_O_3_. These oxides exhibit valuable properties, particularly magnetism. A combination of the mentioned materials leads to multifunctional composite formation with many potential applications, as follows from several studies. However, this area of science is not fully developed. There are still many aspects to be examined. The synthesized composite and its components were analyzed by employing the following methods. The X-ray diffraction analysis revealed formation of hydroxyapatite and Fe_2_O_3_ crystalline phases. Moreover, porosimetry proved a larger specific area for the composite sample in comparison with other materials. The results obtained using the SEM method confirmed an external layer of hydroxyapatite and spherical shapes of internal Fe_2_O_3_ particles. Furthermore, the X-ray photoelectron spectroscopy data presented characteristic peaks of Fe, Ca, P, and O atoms in all samples. The Fourier Transform Infrared spectra displayed all the specific vibrations typical of the analyzed materials. What is more, the Vibrating Sample Magnetometer method confirmed the paramagnetic nature of the samples. It could be concluded that the synthesized composite has intermediate properties between the components used in the formation process. The results suggest that these composites are superparamagnetic. This type of material architecture would be well suited for biomedical applications.

## 1. Introduction

Hydroxyapatite (Hap) is a representative of apatites—a wide group of phosphate minerals naturally occurring in the environment [1]. Its chemical formula is Ca_10_(PO_4_)_6_(OH)_2_. The Ca/P ratio in stoichiometric hydroxyapatite is equal to 1.67, but in nonstoichiometric hydroxyapatite it is between 1.5–2.0 [2]. The compound is a part of human bones and teeth, which makes them properly durable. As a solid, it takes the form of a white powder. Moreover, Hap did not exhibit solubility in water [3]. It does not dissolve at all in bases. It can be concluded that the compound’s ability to dissolve depends on the kind of solvent and the pH value [4,5,6].

Hydroxyapatite is an outstanding biomaterial due to its sorption properties. It contains empty areas in its structure and therefore it can be modified by cationic or anionic substitutions. Hap also owes its popularity to other features, namely its inexpensiveness, ease of synthesis, stability, reactivity, and biocompatibility. Particularly, the latter property makes it widely used in medicine i.e., bone healing processes or implants. What is more, due to the appropriate specific surface area and the ability to bind compounds on its surface, hydroxyapatite can be employed as a drug delivery and release medium. Numerous studies proved that Hap is an effective sorbent in the adsorption processes of metal ions, including heavy metals, as well as in radioactive ions. Furthermore, there is a possibility of adsorption of both inorganic and organic [4,5,6].

Nowadays studies on the potential modifications of hydroxyapatite are becoming more and more popular. The particles, which exhibit magnetic properties, are namely iron oxides and deserve special attention due to their distinguished features, e.g., magnetism and pervasiveness. These oxides exist in various forms and find applications in many branches of industry. Nevertheless, most studies deal with magnetite (Fe_3_O_4_) and maghemite (γ-Fe_2_O_3_), wherein the first one is more common. Magnetite is a mineral with metallic sheen. Its structure is composed of both Fe (II) and Fe (III) with a 1:2 ratio. This mixed oxide displays excellent magnetic properties and electrical conductivity. Fe_3_O_4_ occurs naturally in rocks and can be transformed into other forms. In turn, maghemite is built of Fe (III). This mineral is unstable in the environment and can convert into the hematite form at higher temperatures [7,8,9,10]. The magnetic properties exhibited by iron oxides could be a vital factor that is crucial for any potential possible applications of modified hydroxyapatite, e.g., in medicine or environmental protection.

Materials whose structure is composed of core and shell are becoming more and more popular nowadays [11,12]. Combining specific properties of components, a functional composite could be obtained. Moreover, each synthesis route affects the core and shell characteristics, i.e., the size, shape, or surface. Thus, the composite functionality can be modified, which broadens their potential applications horizon [13].

Specific properties of hydroxyapatite make it a promising coating material due to its large specific area, adsorption capacity, and stability. An additional layer on the iron oxide particles provides better stability, proper diffusion, and protects from aggregation. What is more, such magnetic composites can be easily separated from solution using the external magnet. Combination of the mentioned substrates allows us to obtain a multifunctional material with outstanding features, which can find applications in many branches. Many studies proved that a Hap/iron oxide composite is an excellent adsorbent in metal ions removal processes, e.g., Cd, Cu, Ni, Fe, Mn, Co, U. Moreover, there is a possibility of binding anions on its surface, e.g., nitrates. One of the most vital applications of magnetic hydroxyapatite is medicine. The material could be used as a contrasting agent for the magnetic resonance imaging technique. Its excellent surface properties made it suitable for use as a drug delivery and release medium in the human body. Research shows that various substances could be transported, i.e., drugs, genes, antibiotics, vitamins, hormones, proteins, and enzymes. Furthermore, the composite could find application in the hyperthermia technique, which neutralizes cancer cells. Due to an aging population and increased risk of severe bone fractures, orthopedics became a significant area. Many scientists have worked on developing new and efficient materials that will be durable enough. A promising opportunity is in employing a Hap/iron oxide composite as an implant or rebuilding agent in bone recovery processes. Additionally, the material was investigated for catalytic activity. The studies show that it could be employed as a catalyst in various reactions, e.g., syntheses, oxidation, or degradation [4,14].

As can be seen, there are a lot of studies concerning potential applications of hydroxyapatite with a magnetic core. However, most research covers the synthesis of Hap and the Fe_3_O_4_ core. There is still a need to broaden this topic, particularly concerning the γ-Fe_2_O_3_ core. Moreover, it has not been fully investigated for adsorption—only selected ions were examined. This area still has a lot to explore.

This paper focuses on the synthesis of the hydroxyapatite/iron oxide (γ-Fe_2_O_3_) composite utilizing the co-precipitation method and the analysis of selected properties.

## 2. Materials and Methods

The reagents used in this research are the following: (NH_4_)_2_HPO_4_ (POCh, analytical grade reagent), Ca(NO_3_)_2_·4H_2_O (POCh, analytical grade reagent), NH_4_OH (Chempur, analytical grade reagent), Fe_2_O_3_ (Alfa Aesar), NaOH (POCh, analytical grade reagent), HCl (POCh, analytical grade reagent), and NaCl (POCh, analytical grade reagent).

The samples were analyzed by means of the following methods. Employing the ASAP2405 apparatus (Micromeritics Inc., Norcross, GA, USA), the specific surface area and porosity were measured using the nitrogen adsorption/desorption isotherms. The ambient temperature was 22 °C, the degassing temperature was 50 °C, and the bath temperature was equal to −195.85 °C for each sample. The crystal structure was studied by the X-ray diffractometer (XRD) (Empyrean, PANalytical, Malvern, UK). The minimum step size for adjusting the incidence angle and the scatter angle was 0.0001 degrees. The surface was also characterized by scanning electron microscopy (SEM) utilizing the QUANTA 3D FEG apparatus Oregano, USA. Furthermore, the physicochemical properties of the obtained samples were analyzed with X-ray photoelectron spectroscopy (XPS) (MX-650, Gammadata Scienta, Uppsala, Sweden). Fourier Transform Infrared (FTIR) spectra were also obtained employing the Nicolet 8700A apparatus (Thermo Scientific, San Jose, CA, USA). The particle size was calculated using the laser diffraction method (Mastersizer 2000, Malvern Instruments, Malvern, UK). Moreover, potentiometric titration was used for the surface charge measurement. The analysis was conducted in a Teflon vessel with a propeller stirrer. The temperature consistency was maintained by a thermostat (LaudaRE204, Germany). An inert atmosphere was sustained utilizing gaseous nitrogen. A measuring system was built of an automatic burette (Dosimat 665, Metrohm, Singapore), pH meter (PHM-240, Radiometr, Copenhagen, Denmark), indicator electrode (pHG201-8, Radiometr, Copenhagen, Denmark), and reference electrode (REF 451, Radiometr, Copenhagen, Denmark). The instruments were connected by means of the computer program. Furthermore, the zeta potential was measured (Zetasizer Nano ZS90, Malvern, UK). The suspension was prepared by ultrasonication of 0.01 g of the solid sample. The magnetic properties of the samples were studied over the temperature range of 4–300 K at the magnetic field 0.1 T using a Quantum Design SQUID-VSM (Vibrating Sample Magnometer, Darmstadt, Germany). The gram susceptibility was investigated to get to know whether the nature of the magnetic interactions changed at low temperatures. The field dependences of magnetization were investigated at 77 K and at room temperature in the applied field up to 5 T and were corrected by subtracting the sample–holder signal and the contribution χD estimated from Pascal’s constants [15].

### 2.1. Hydroxyapatite Synthesis

Hydroxyapatite powder was obtained using the co-precipitation method. Briefly, 200 mL of 0.75 M Ca(NO_3_)_2_·4H_2_O was placed in a three-necked flask and heated to 100 °C for 30 min by employing a water bath with continuous stirring. Then, 200 mL of 1 M (NH_4_)_2_HPO_4_ was dropped for 2 h. The pH value of the added solution was previously adjusted to the value of 10 utilizing NH_4_OH. Finally, the obtained precipitate was rinsed and centrifuged using distilled water until the conductivity value was stable. Finally, the white powder was dried in the oven at 100 °C for 24 h.

### 2.2. Composite Formation

The composite formation proceeded in the same procedure as the hydroxyapatite. The co-precipitation method was also employed. Briefly, the 200 mL of 0.75 M Ca(NO_3_)_2_·4H_2_O solution and 0.77 g of iron (III) oxide were put into the three-necked flask. The mixture was heated to 100 °C using the water bath and stirred continuously for 30 min. Then, 200 mL of 1 M (NH_4_)_2_HPO_4_ was dropped for 2 h. Next, the obtained powder was rinsed and centrifuged using distilled water until the conductivity value was stable. Finally, the precipitate was dried in the oven at 100 °C for 24 h.

## 3. Results and Discussion

### 3.1. Surface Analysis by the Porosimetry Method

Table 1 presents the collected data. It can be seen that the composite has a larger surface area than its components, which improves its adsorption capacity. Moreover, the pore size varies; there are mesopores everywhere. Those of the composite are the largest. There was a measurement accuracy 0.03% and a measurement repeatability ± 0.01%.

### 3.2. Phase Identification via Powder X-ray Diffraction

**Figure 1:**
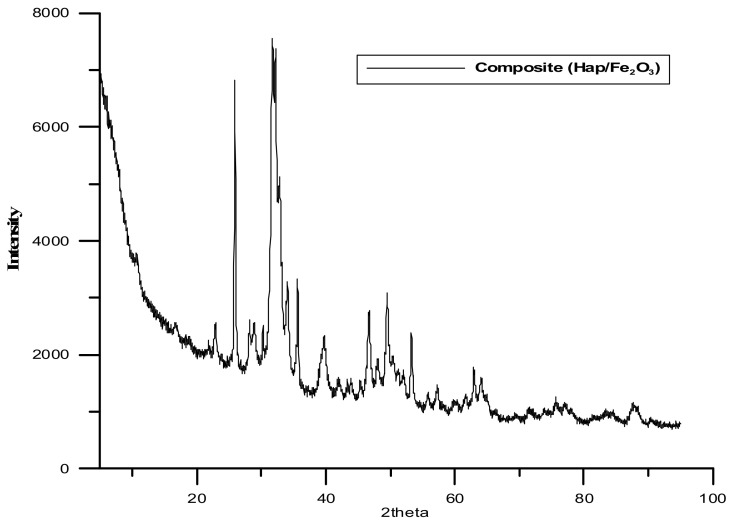
XRD spectrum of the composite.

**Figure 2:**
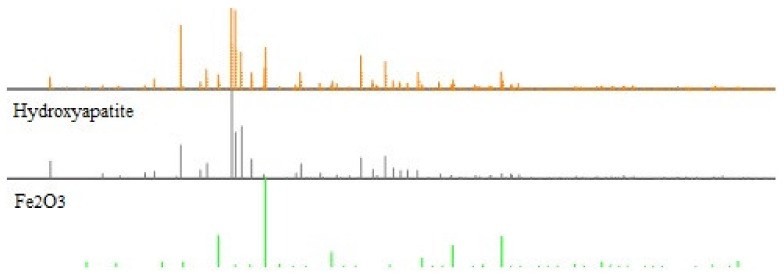
The XRD analysis of the composite [16].

The next analysis employed the XRD method (X-ray Diffraction). Table 2 and Figure 1 and Figure 2 present the obtained results. It is worth mentioning that the formation of the composite does not result in the formation of a new crystalline form. The diffraction pattern represents a well crystallized single-phase hydroxyapatite with a hexagonal structure. The XRD peaks around 2θ: 25.9°, 29.1°, 31.7°, 32.7°, 39.8°, 49.4°, and 53.0° correspond to the reflection from the (002), (102), (211), (112), (300), (202), (310), (213), and (004) crystal planes of hydroxyapatite, respectively. The diffraction pattern of the composite shows dual phases, hydroxyapatite and Fe_2_O_3_. The X-ray pattern of the obtained iron trioxide nanoparticles indicates the creation of γ-Fe_2_O_3_, which is proved by the following peaks: 35.55, 62.6, 44.5, and 50. Many sharp peaks were observed indicating that the samples have high crystallinity.

The crystallite sizes were calculated from the XRD data using Scherer.

D = 0.9λ/(β Cos θ)

where:

- D is the crystallite size,
- λ is the wavelength of X-ray used (1.5406 Å),
- β is the value of peak half width.

FWHM (radians) and θ is the angle of diffraction. Moreover, the crystallite sizes were calculated for the hydroxyapatite and iron (III) oxide phases. They were equal to 118 Å and 613 Å, respectively.

### 3.3. SEM

**Figure 3:**
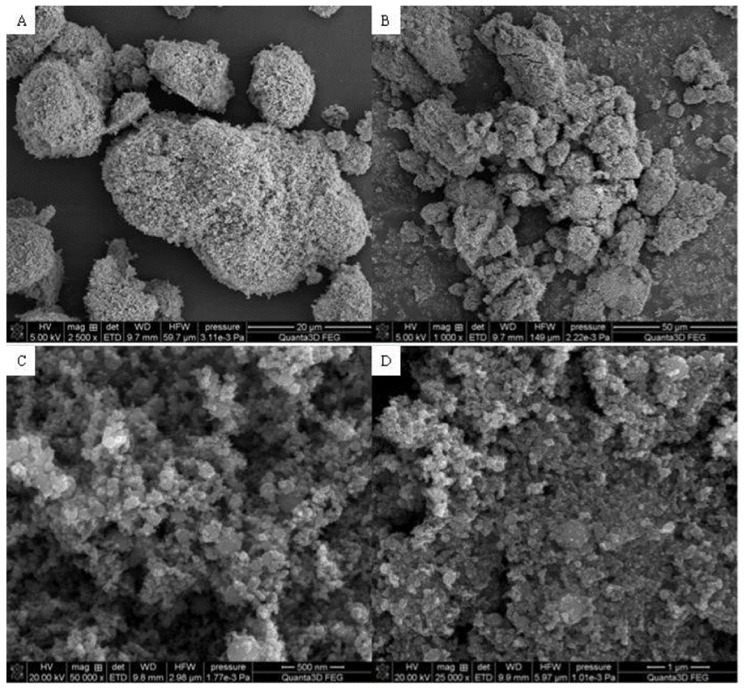
SEM photographs of Fe_2_O_3_ (**A**) 20 μm, (**B**) 50 μm, (**C**) 500 nm, (**D**) 1 μm.

**Figure 4:**
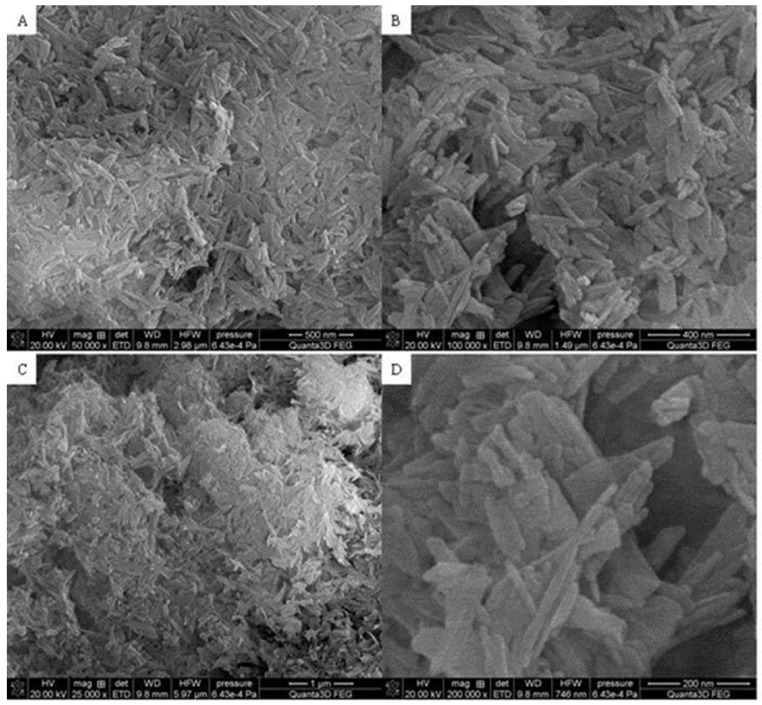
SEM photograph of Hap (**A**) 500 nm, (**B**) 400 nm, (**C**) 1 μm, (**D**) 200 nm.

**Figure 5:**
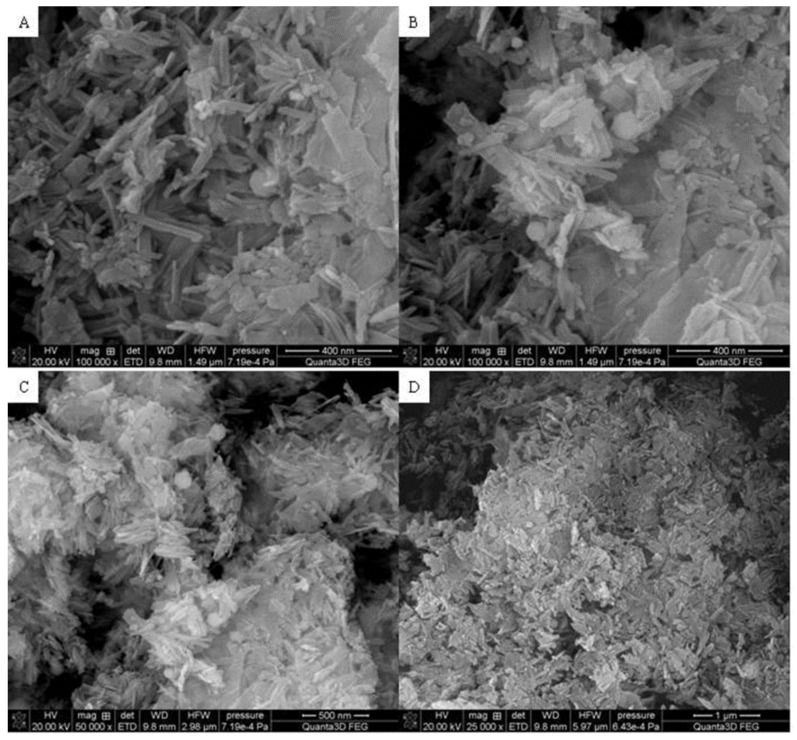
SEM photograph of the composite (**A**) 400 nm, (**B**) 400 nm, (**C**) 500 nm, (**D**) 1 μm.

Figure 3, Figure 4 and Figure 5 display the SEM photographs of the analyzed materials surface. Figure 3 presents the Fe_2_O_3_ sample. The particles have spherical shapes and various sizes. It can be seen that larger agglomerates are formed. The structure seems to be porous with sparse tubules. The Hap surface with flat, cylindrical shapes is shown in Figure 4. The particles are regular and connected with each other. Furthermore, Figure 5 exhibits the composite sample. The part derived from Hap and Fe_2_O_3_ can be distinguished. Hydroxyapatite forms the external layer where molecules have a characteristic cylindrical shape. Spherical iron oxide particles are mostly covered with Hap but some of them can be seen scattered on the surface. In general, the synthesized composite is heterogeneous with the empty areas. The SEM photographs revealed an agglomerate of particles at the surface, which appeared compacted and coarse. The result also revealed some level of porosity on the surface, offering the material a good adsorption characteristic.

### 3.4. X-ray Photo-Electron Spectroscopy (XPS)

**Figure 6:**
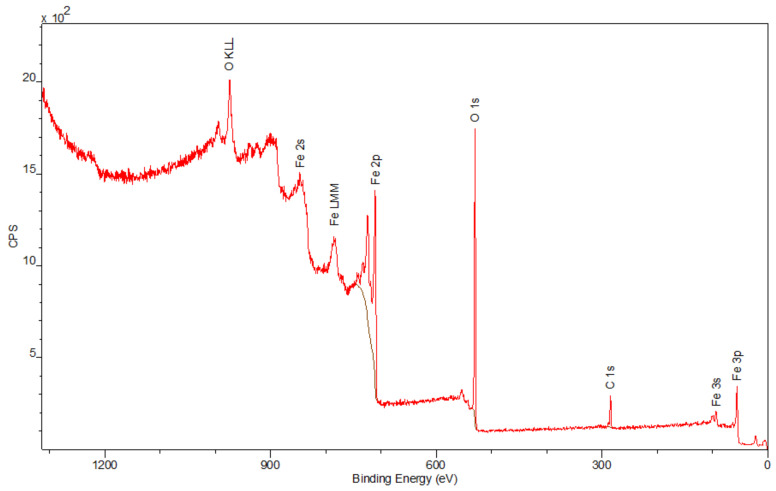
The XPS spectrum of Fe_2_O_3_.

**Figure 7:**
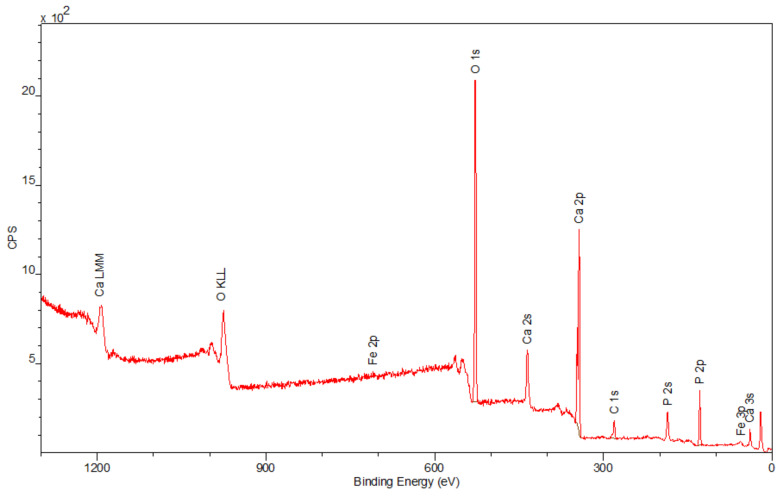
The XPS spectrum of Hap.

**Figure 8:**
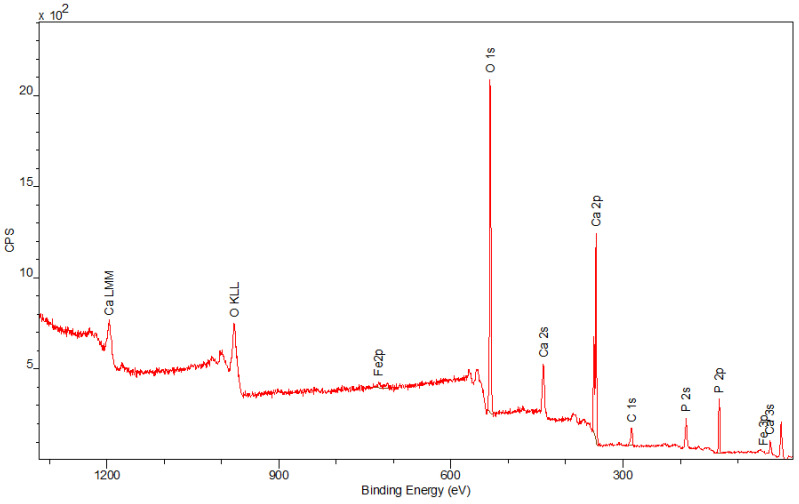
The XPS spectrum of the composite.

The spectra obtained by the XPS method are shown in Figure 6, Figure 7 and Figure 8. Besides the expected Fe (2p) and O (1s) peaks for the Fe_2_O_3_ sample; Ca (2p), P (2p), and O (1s) for the Hap sample; and Fe (2p), O (1s), Ca (2p), and P (2p) for the composite sample, the peak C (1s) was also observed in all mentioned materials. Its presence in the examined compounds varies from 4.22% to 7.86% and can be caused by the adsorption of impurities from the atmosphere. Due to Fe_2_O_3_ being covered with hydroxyapatite in Figure 8, a smaller intensity at the Fe (2p) peak is observed compared to Figure 6. The analysis confirmed the presence of Fe (II) and Fe (III). The intensities of the Ca (2p) and P (2p) peaks in the Hap and its combination with iron oxide are similar. The Ca/P ratio calculated for the composite is equal to 1.58, which indicates that the obtained material is non-stoichiometric. The peak at 531.2 eV in Figure 8 indicates O (1s), which can also be seen in the Fe_2_O_3_ and Hap spectra. In the studied samples, oxygen is combined with metal oxide, hydroxide, and phosphate. There are no significant differences between the components’ spectra and composite spectrum.

### 3.5. IR Analysis

**Figure 9:**
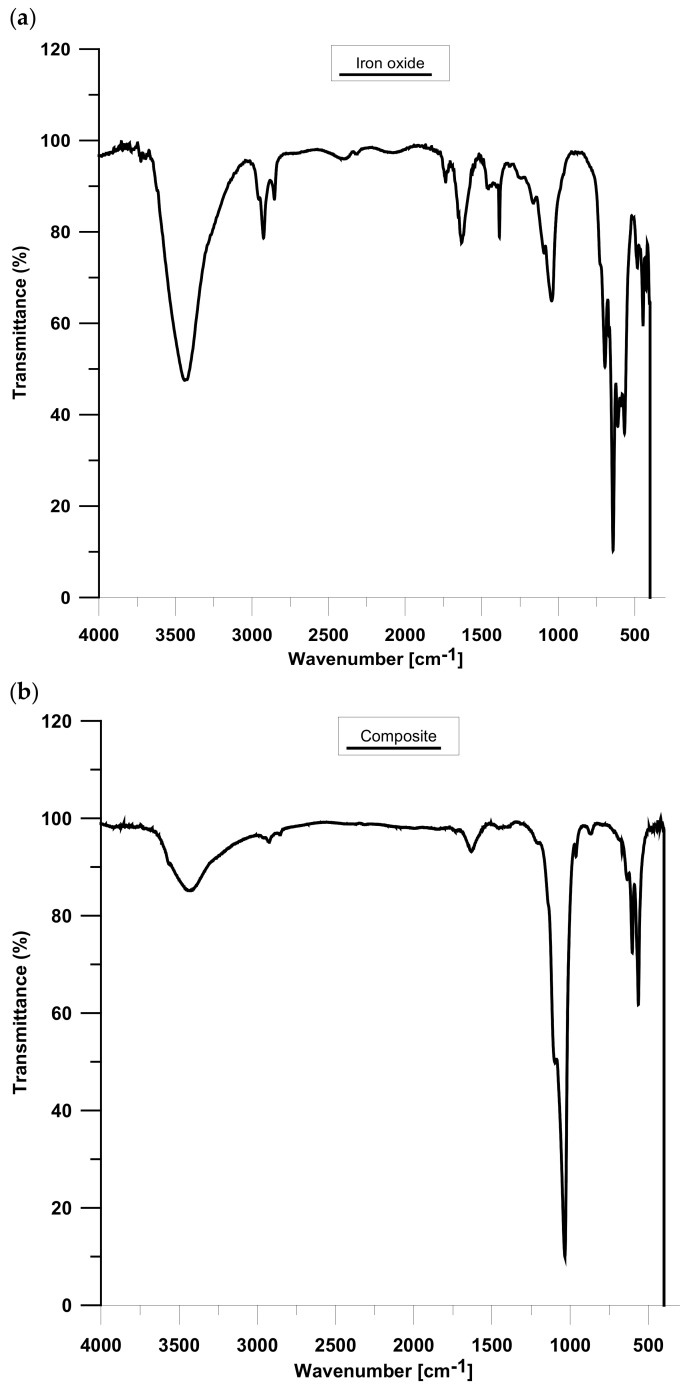
FTIR analysis of (**a**) iron oxide and (**b**) composite.

Figure 9 displays the results of the IR analysis of iron oxide (A) and composite (B). The A spectrum proved the presence of maghemite particles, as follows from the characteristic peaks from 800 to 400 cm^−1^, which could be attributed to spinel γ-Fe_2_O_3_ [17]. Moreover, the band at 591 cm^−1^ is related to the vibrations of γ Fe-O and the peaks at 567 and 695 cm^−1^ can be assigned to γ-Fe_2_O_3_ [18,19]. Due to the literature data, the band at 632 cm^−1^ is characteristic of maghemite. Moreover, magnetite does not have any specific peaks over 600 cm^−1^ [20]. Thus, this is evidence of successful γ-Fe_2_O_3_ formation. Although the XPS method showed the presence of Fe (II), however it could be oxidized to the Fe (III) form, characteristic peaks of Fe_3_O_4_ are not observed on the IR spectrum [20]. Furthermore, the bands at 3430 cm^−1^ and 1631 cm^−1^ can be related to the stretching and bending vibrations of the –OH group of absorbed water [18]. The composite spectrum also has peaks attributed to H_2_O, i.e., 3424 and 1631 cm^−1^ [18]. The bands at 1034 and 963 cm^−1^ are related to the stretching vibrations of the phosphate group. Additionally, the bending vibrations of the mentioned group are observed at 603 and 564 cm^−1^ [18,21,22]. The vibrations of PO_4_^3−^ confirmed the presence of the hydroxyapatite outer layer. Furthermore, the obtained material is contaminated with carbonate groups derived from atmospheric CO_2_, which can be observed at 870 cm^−1^ [18,23]. Moreover, the additional organic impurities can be seen in the range of 2900–2800 cm^−1^.

### 3.6. Particle Size Measurements

The particle size analysis of the samples of hydroxyapatite, iron oxide, and composite conditioned in electrolyte solutions allows for the discussion of the aggregation/dispersion processes of hydroxyapatite, iron oxide, and composite particles, or the process of precipitation of a new phase in hydroxyapatite, iron oxide, and composite suspension on the basis of changes in the particle distribution Table 3.

The monomodal model was used in the apparatus to determine the particle size. The model size found where the frequency distribution reaches a maximum. If the frequency distribution has only one maximum, this is called monomodal. From the results we can see that the pH of the tested systems changes after 7 days of conditioning in the solution. For the hydroxyapatite sample, it decreases from 7.37 to 6.87; in the composite from 7.31 to 6.99, most likely due to leaching of anionic impurities after synthesis from the pores; and for iron oxide the pH changes from 5.31 to 6.35, most likely due to leaching of cationic impurities after synthesis from the pores. The average particle size of hydroxyapatite varied from 4.155 µm to 4.597 µm, iron oxide from 1.819 µm to 2.268 µm, and the composite from 7.802 µm do 7.984 µm. The appearance of hydroxyapatite particle aggregates can be partially explained by the slow coagulation phenomenon, although the electrokinetic potential is small. It seems that the process of aggregation may be supported by the processes of hydroxyapatite dissolution and the precipitation of the new phase with the participation of Cl ions.

### 3.7. Surface Charge Measurements

**Figure 10:**
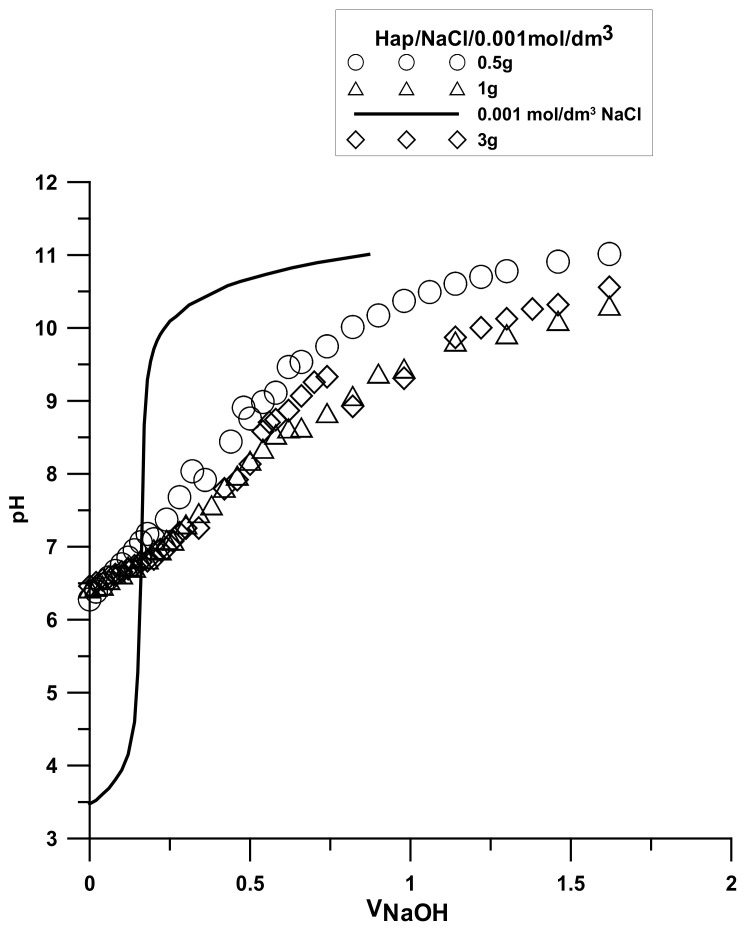
Titration curve for the hydroxyapatite sample.

**Figure 11:**
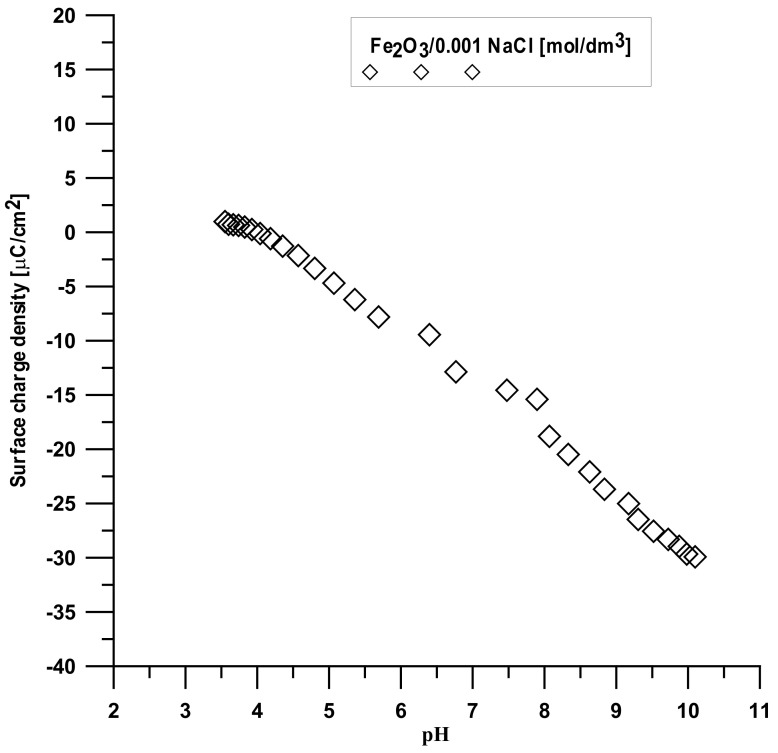
Titration curve for the Fe_2_O_3_ sample.

**Figure 12:**
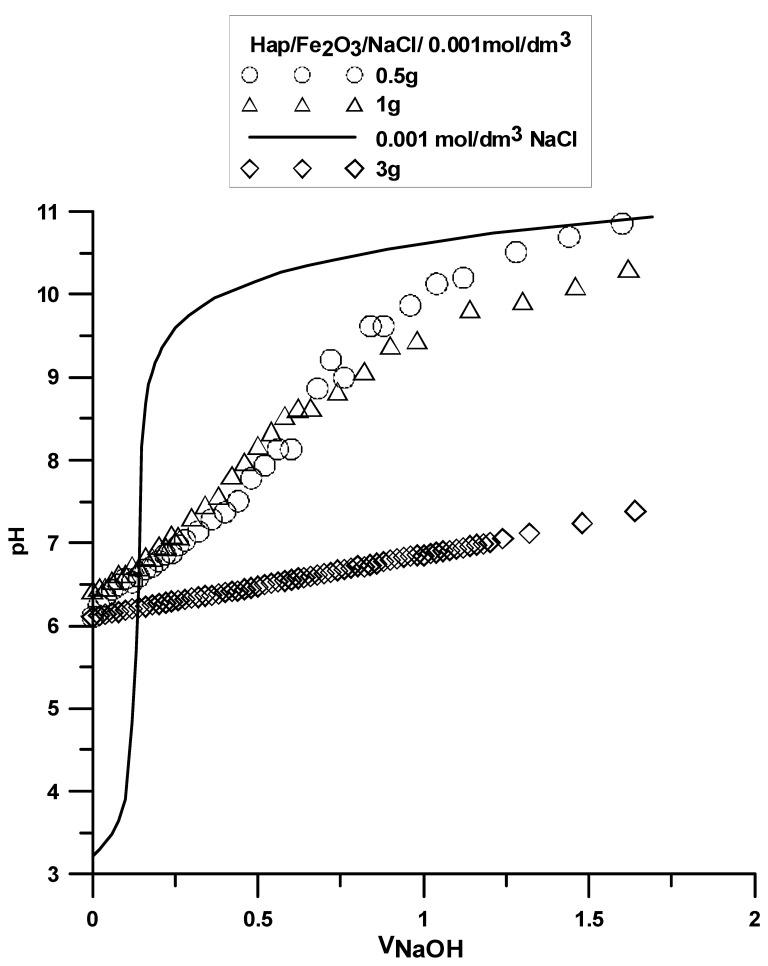
Titration curve for the Hap/Fe_2_O_3_ composite sample.

The analysis was made based on the potentiometric titration method. In order to determine the point of zero charge (pHpzc) for hydroxyapatite and the composite, three titrations with different weights of powders were made. For iron (III) oxide, the titration was only performed for one sample. The titration curves for each sample are presented in Figure 10, Figure 11 and Figure 12. The intersection point of the obtained titration curves and the electrolyte curve was determined, which led to finding the point of zero charge. The point of the curve’s intersection corresponds to the pH, at which there is the point of zero charge for hydroxyapatite being equal to seven and approximately sic for the composite. The pHpzc for Fe_2_O_3_, determined in a different way due to the smaller solubility of the compound, is four.

There is a clear difference in this value between the components of the composite and the composite itself. The shift of this value towards hydroxyapatite results from the dominant role of the phosphate and calcium groups on the composite’s surface.

**Figure 13:**
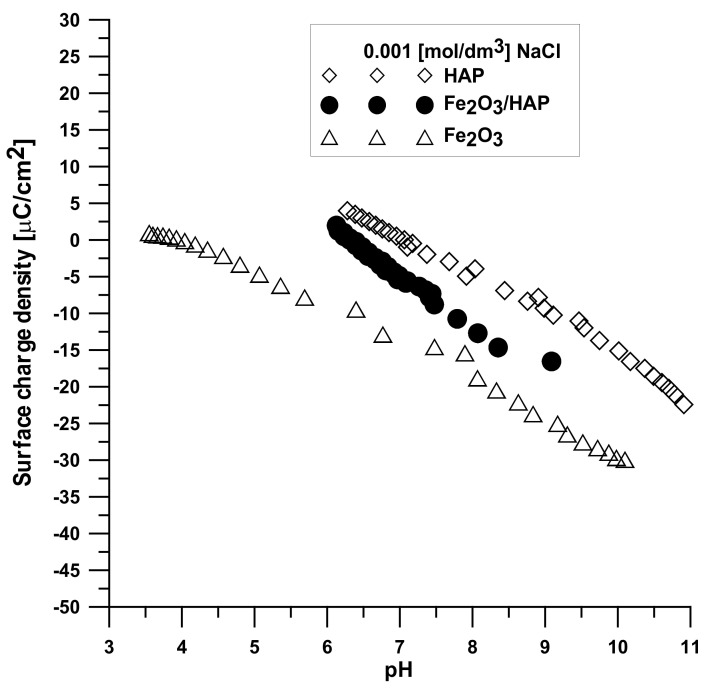
Dependence of surface charge density on the pH value.

Figure 13 displays the dependence of the surface charge density on the pH value for each compound. It can be seen that the increase in the pH values causes a change in the surface charge density, especially after pHpzc, which is associated with the release of ions from the adsorbent surface.

### 3.8. Electrophoretic Mobility Measurements Converted to the Zeta Potential

**Figure 14:**
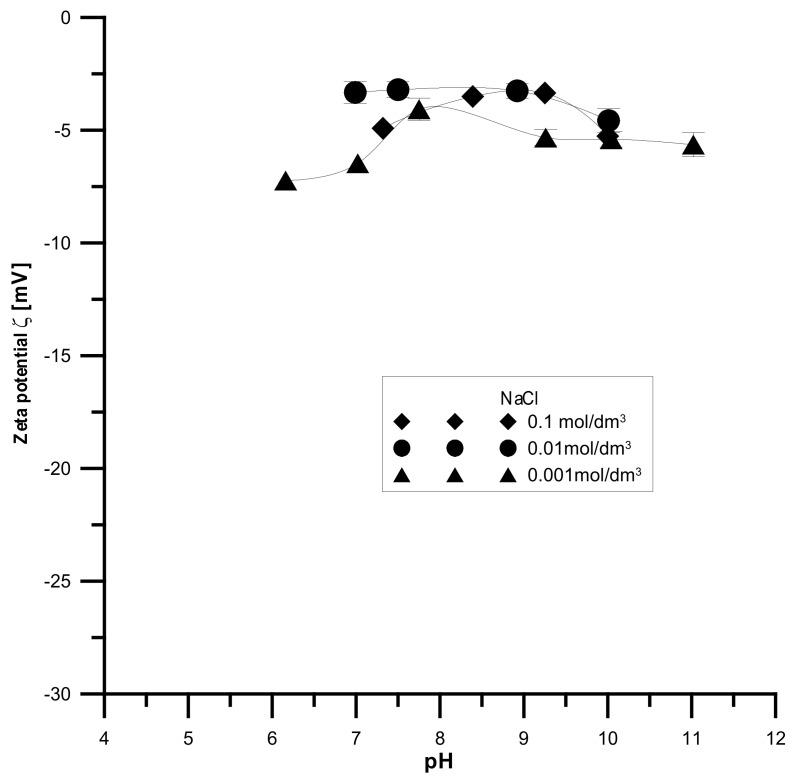
Relationship between the pH value and the zeta potential for the HAP sample.

**Figure 15:**
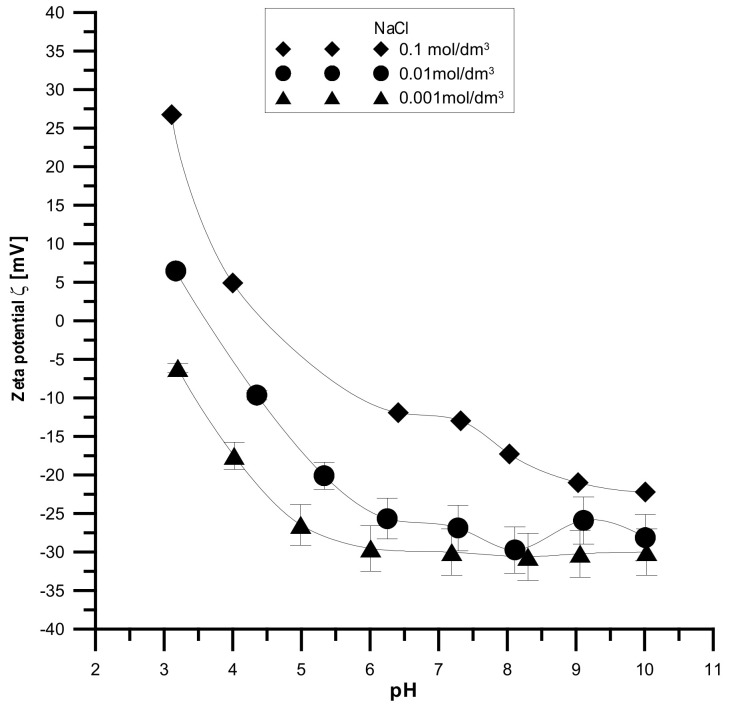
Relationship between the pH value and the zeta potential for the Fe_2_O_3_ sample.

**Figure 16:**
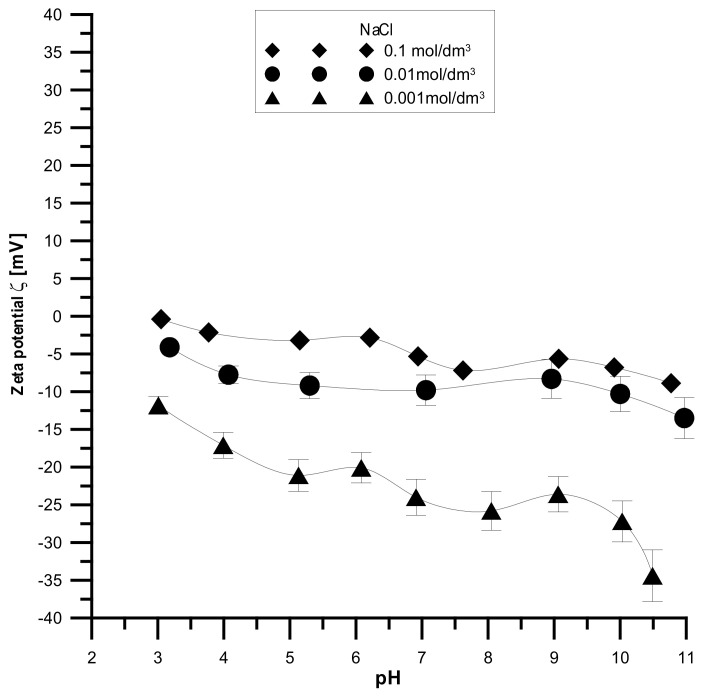
Relationship between the pH value and the zeta potential for the composite sample.

The analyzed samples have the highest absolute values of the zeta potential for the smallest electrolyte concentration and the lowest absolute values for the smallest electrolyte concentration, which is shown in Figure 14, Figure 15 and Figure 16. It was found that the zeta potential decreases with an increase in the electrolyte concentration resulting from the surface group’s dissociation. It can also be seen that the zeta potential decreases with the increasing pH. The zeta potential was studied in the pH range from 3 to 11, and the zeta potential range was from 30 mV to −35 mV, which indicates that the samples are unstable in the colloidal form, and they can delaminate. Only the Fe_2_O_3_ sample in the 0.001 M NaCl solution where the pH was in the range of 8–10 was stable. Additionally, the composite samples showed stability only in the greatest ranges of pH. However, hydroxyapatite in the whole range proved unstable. The comparison of the point of zero charge and the isoelectric point indicates that the isoelectric point is shifted towards acids and is less than three for all samples.

Moreover, the analyzed samples are porous. Therefore, a large part of the charge can be compensated inside the pores, i.e., the voids between the particles in the aggregates, and only the part coming from the ionized groups on the outer surface can be responsible for electrophoretic mobility.

### 3.9. VSM

**Figure 17:**
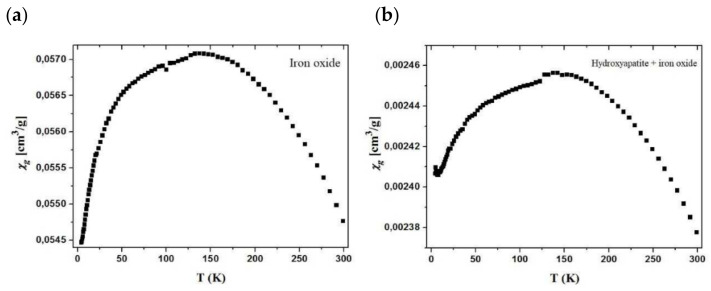
Magnetization characterization of the analyzed materials, (**a**) pure oxide, (**b**) hydroxy-apatite material.

The obtained values prove the paramagnetic nature of the samples. The relationships of χg values vs. T for the samples are shown in Figure 17. From the χg vs. T curve course, it appears that the χg values increase as the temperature drops to about 150 K. This may suggest ferromagnetic intermolecular interactions. While the samples are cooled down further to 4 K, the χg values decrease from 0.0571 cm^3^/g to 0.0547 cm^3^/g for pure oxide (Figure 17a) and 0.00246 cm^3^/g to 0.00238 cm^3^/g for the hydroxyapatite material (Figure 17b). However, in the case of pure oxide, these changes are more rapid as opposed to the modified material where they diminish slowly, revealing the saturation paramagnetic state. The fluctuations in the χg value indicates positive and negative θ values, which may suggest the ferromagnetic and antiferromagnetic intermolecular interactions depending on the temperature. Below the temperature of 150 K, the material shows antiferromagnetic interactions, which are visible on the graph (decrease in gram compliance value), and its value nevertheless continues to be positive, indicating the magnetic nature of the sample. Furthermore, the antiferromagnetic interactions only reduce its value.

### 3.10. The Comparison of Employed Synthesis Method and Other Approaches

In comparison with the hydrothermal method, the results were comparable. However, the approach conducted by Govindan et al. required specialized equipment and high temperatures and pressures [24]. These conditions are rather aggressive. Moreover, the hydrothermal technique is quite long, and the formed particles could grow uncontrollably. Furthermore, the main advantage of the chemical precipitation method over the sol–gel method presented by Yusoff et al. [25] is the fact that in the second one there is a significant risk of synthesized material cracking during the drying process.

There are many possible routes to synthesize hydroxyapatite with a magnetic core. Each approach has a specific effect on the material’s properties. For example, Mondal et al. showed different shapes that the composite could have, i.e., nut-shaped, sheets, spherical, nanotubes, rice shapes, and rods [26]. However, the greatest advantages of the chemical precipitation method over the rest of the techniques are its simplicity, low cost, and eco-friendliness, which is particularly significant these days due to “green” chemistry principles.

## 4. Conclusions

To conclude, the selected synthesis method turned out to be an effective method and, in addition, it did not require great financial costs. The XRD study of the crystalline structure of the composites revealed the presence of the crystalline phase of hydroxyapatite and Fe_2_O_3_. The precipitation of Hap on Fe_2_O_3_ changes the conditions for Hap formation as compared to Hap formation itself. This difference affects the surface properties of the obtained materials, which can be seen in the porosimetry analysis. The photographs gathered from the SEM method show the formation of the Hap external layer and the spherical particles of Fe_2_O_3_, which are covered. The XPS method confirmed the presence of Fe, Ca, P, and O atoms in all samples, as is shown by the characteristic peaks. The IR spectra proved the successful formation of a hydroxyapatite outer layer and iron (III) oxide core. The VSM analysis exhibited the paramagnetic nature of the samples. Moreover, as follows from the comparative studies, in most cases the composite is characterized by the properties of the intermediate between the hydroxyapatite and Fe_2_O_3_ used for the synthesis. The obtained composite shows satisfactory properties, confirming the effectiveness of the syntheses. The results suggest that these composites are superparamagnetic. This type of material architecture would be well suited for biomedical applications.

## Figures and Tables

**Table 1:** Porosity measurements.

	Hap	Fe_2_O_3_	Fe_2_O_3_/Hap
Surface area from the BET isotherm [m^2^/g]	81	31	82
Surface area from the Langmuir isotherm [m^2^/g]	118	48	123
Total pore volume from adsorption 1.7 nm < d < 300 nm by the BJH method [cm^3^/g]	0.37	0.10	0.44
Total pore volume from desorption 1.7 nm < d < 300 nm by the BJH method [cm^3^/g]	0.37	0.10	0.44
Average pore radius from the BET method [nm]	18.63	12.72	21.26
Average pore radius from the BJH adsorption method [nm]	18.81	14.51	19.37
Average pore radius from the BJH desorption method [nm]	17.50	14.05	17.95

**Table 2:** The composite analysis by the XRD method.

Compound Name	Chemical Formula	Quantitative Share (%)
Calcium phosphate hydroxide	Ca_5_(PO_4_)_3_OH	97.5 (5)
Iron (III) oxide	Fe_2_O_3_	2.47 (8)

**Table 3:** Particle size measurements.

Change in the Particle Size Distribution over Time		Analyzed Substance in the Solution of 0.001 M NaCl		
		Hydroxyapatite	Composite	Iron (III) Oxide
0 h	pH	7.37	7.31	5.31
	μm	4.155	7.802	1.819
1 h	μm	4.225	7.806	1.915
3 h	μm	4.241	7.819	1.924
24 h	μm	4.275	7.826	2.070
48 h	μm	4.298	7.838	2.182
5 days	μm	4.303	7.930	2.121
7 days	μm	4.597	7.984	2.268
	pH	6.87	6.99	6.35

## Data Availability

The data underlying this article will be shared on reasonable request from the corresponding author.
